# Annual acknowledgement of manuscript reviewers

**DOI:** 10.1186/1824-7288-40-15

**Published:** 2014-02-13

**Authors:** Sergio Bernasconi, Carlo Caffarelli, Francesca Santamaria

**Affiliations:** 1 Editor-in-Chief, Italian Journal of Pediatrics, University of Parma, Parma, Italy; 2 Pediatric Department, Azienda Ospedaliera-Universitaria, University of Parma, Parma, Italy; 3 Pediatric Department, "Federico II" University, Naples, Italy

## Abstract

**Contributing reviewers:**

The editors of *Italian Journal of Pediatrics* would like to thank all our reviewers who have contributed to the journal in Volume 39 (2013).

## 

Samuel Adegoke

Nigeria

Walter Ageno

Italy

Carlo Agostoni

Italy

Luísa Aires

Portugal

Hisaya Akiba

Japan

Manuela Albisetti

Switzerland

Maria Alessio

Italy

Omar Alkandari

Canada

Sergio Amarri

Italy

Tarek Amin

Saudi Arabia

Hisami Ando

Japan

Jesús Argente

Spain

Simona Asole

Italy

Sarah Atkinson

United Kingdom

Massimo Attanasio

United States of America

Raffaele Badolato

Italy

Rossana Bagna

Italy

William Baine

United States of America

Mustafa Bakir

Turkey

Paolo Balestri

Italy

Umberto Balottin

Italy

Oya Baltali Halicioglu

Turkey

Siddharth Banka

United Kingdom

Sriparna Basu

India

Scot Bateman

United States of America

Shahin Behjati

Iran

Hassan Bella

Saudi Arabia

Joseph Bellanti

United States of America

Carlo Bellieni

Italy

Alberto Berardi

Italy

Roberto Berni Canani

Italy

Federica Berrilli

Italy

Eugenia Bezirtzoglou

Greece

Gianni Bisogno

Italy

Carla Bizzarri

Italy

Alfredo Boccaccino

Italy

Osvaldo Borrelli

United Kingdom

Arend Bos

Netherlands

Ugo Bottone

Italy

Sofia Bouhlal

United States of America

Rodolfo Bracci

Italy

Paolo Brambilla

Italy

Carlo Brera

Italy

Itzhak Brook

United States of America

Nicola Brunetti-Pierri

Italy

Alessandra Buja

Italy

Giuseppe Buonocore

Italy

Craig Burkhart

United States of America

Fabio Buzi

Italy

Carlo Caffarelli

Italy

Inês Camacho

Portugal

Angelo Campanozzi

Italy

Andrew Campbell

United States of America

Mario Canciani

Italy

Fuat Emre Canpolat

Turkey

Nicola Carano

Italy

Francisco Cardoso

Brazil

Carlo Catassi

Italy

Anthony Catto-Smith

Australia

Claudio Cavalli

Italy

Luciano Cavallo

Italy

Atilla Cayir

Turkey

Franco Cerutti

Italy

Elena Chiappini

Italy

Francesco Chiarelli

Italy

Gaetano Chirico

Italy

PoHan Chou

Taiwan

Carlo Cianchetti

Italy

Javier Cieza Zevallos

Peru

Maria Roberta Cilio

United States of America

Marta Luisa Ciofi degli Atti

Italy

Fabris Claudio

Italy

Antonio Clavenna

Italy

William Collins

United States of America

Raffaella Colombatti

Italy

Daniela Concolino

Italy

Alessandro Consolaro

Italy

Roberto Copetti

Italy

Luigi Corvaglia

Italy

Salvatore Cucchiara

Italy

Paolo Curatolo

Italy

Renato Cutrera

Italy

Bruno Dallapiccola

Italy

Albane Dandolo

France

Carlo Dani

Italy

Philip Davies

United Kingdom

Daniele De Luca

Italy

Maurizio de Martino

Italy

Giuseppe De Nisi

Italy

Vincenzo De Sanctis

Italy

Jean de Ville de Goyet

Italy

Fabio Decimo

Italy

Ennio Del Giudice

Italy

Iride Dello Iacono

Italy

Giovanni Di Nardo

Italy

Carlo Dionisi-Vici

Italy

Arianna Dondi

Italy

Andrea Dotta

Italy

Therese Dowswell

United Kingdom

Saskia Drösler

Germany

Junbao Du

China

Marzia Duse

Italy

Sourabh Dutta

India

Martin Edwards

United Kingdom

Enrica Eiva

Italy

Ahmed Elhassanien

Kuwait

Susanna Esposito

Italy

Emilia Falcone

United States of America

Fernando Ferrero

Argentina

Giovanni Battista Ferrero

Italy

Josep Figueras-Aloy

Spain

Marion Flechtner-Mors

Germany

Alberto Flores d'Arcais

Italy

Adriana Franzese

Italy

Neil Frazer

United Kingdom

Izumi Fukuda

Japan

Giancarlo Gargano

Italy

Soeren Gatermann

Germany

Simona Gatti

Italy

Aneta Gawlik

Poland

Diego Gazzolo

Italy

Karla Georges

Trinidad and Tobago

Carolien FM Gijsbers

Netherlands

Ozlem Goker-Alpan

United States of America

W. Christopher Golden

United States of America

Mary Beth Gordon

United States of America

Paul Govaert

Belgium

Frank Greer

United States of America

Nella Augusta Greggio

Italy

Elena Grigorenko

United States of America

Carley Grimes

Australia

Ornella Guardamagna

Italy

Mehmet Haciyanli

Turkey

Larry G.P. Hadley

South Africa

Ali Akbar Haghdoost

Iran

Thomas Hale

United States of America

Hazen Ham

United States of America

Ruth Hamilton

Uruguay

Cathy Hammerman

Israel

Rasha Hamza

Egypt

Malinda Harris

United States of America

Philip Hashkes

Israel

Nevin Hatipoglu

Turkey

Piotr Heczko

Poland

Maria Henwood-Finley

United States of America

Susan Hillier

Australia

William Holmes

United Kingdom

Joost Hopman

Netherlands

Kristina Hulten

United States of America

Santosh Hunasgi

India

Tetsuya Inazu

Japan

Lorenzo Iughetti

Italy

Cecilie Javo

Norway

Marc Jeschke

Canada

Harrison Jones

United States of America

Rishi Kafle

Nepal

Konstantinos Kamperis

Denmark

Mehmet Kantar

Turkey

Paraskevi Karagianni

Greece

Sunil Karande

India

William Keenan

United States of America

Roya Kelishadi

Iran

Ann Kennedy-Behr

Germany

Alison Kesson

Australia

Mohammad Khan

Korea, South

Ali S. Khashan

Ireland

Rosita Kiudeliene

Lithuania

Dwight Koeberl

United States of America

Jun Kohyama

Japan

Yoko Komada

Japan

Bhushan Kumar

India

Przemko Kwinta

Poland

Mario La Rosa

Italy

Richa Lal

India

Massimo Landi

Italy

Susanne Lau

Germany

Virginie Laurier

France

Dariusz Lebensztejn

Poland

Anja Lee

Norway

Vincenzo Leuzzi

Italy

Andrea Lo Vecchio

Italy

Enrico Lombardi

Italy

Riccardo Longhi

Italy

Adriana Lopez

United States of America

Enrico Lopriore

Netherlands

Worawit Louthrenoo

Thailand

Jean Lowe

United States of America

Ernesto Maddaloni

Italy

Rohan Maharaj

Trinidad and Tobago

Raffaele Manna

Italy

Paola Marchisio

Italy

Gian Luigi Marseglia

Italy

Alberto Martini

Italy

Klara Marton

United States of America

Prasad Mathew

United States of America

Paolo Matricardi

Italy

Simone Maurea

Italy

Daniela Melis

Italy

Luigi Memo

Italy

Ornella Milanesi

Italy

Mathieu Milh

France

Lynne Millar

Australia

Roberto Miniero

Italy

Michele Miraglia del Giudice

Italy

Rebecca Mitchell

Australia

Noriko Miyake

Japan

Saeed Mohammad

United States of America

Angelika Mohn

Italy

Diego Moliner-Urdiales

Spain

Giovanni Montini

Italy

Iris Morag

Israel

Fakhrossadat Mortazavi

Iran

Viviana Moschese

Italy

Mohammad Ashkan Moslehi

Iran

Chikako Motomura

Japan

Antonio Muscari

Italy

Jennifer Muszynski

United States of America

Kenneth Myers

Canada

Cristiana Nascimento-Carvalho

Brazil

Mitra Naseri

Iran

Gerard Ngueta

Canada

Felix Niggli

Switzerland

Giovanni Nigro

Italy

Lisbeth Nilsson

Sweden

Michael Noetzel

United States of America

Hedvig Nordeng

Norway

Paul Norman

United Kingdom

Lucia Dora Notarangelo

Italy

Elsebet Oestergaard

Denmark

Rei Ogawa

Japan

Tinuade Ogunlesi

Nigeria

Salvatore Oliva

Italy

Mike Oliver

Australia

Arnold Oranje

Netherlands

Joyce Owens

United States of America

Giovanni Pajno

Italy

Cristina Palacios

Puerto Rico

Frederick Palmer

United States of America

Emmanouil Paraskakis

Greece

Stefano Parmigiani

Italy

Nicholas Passalacqua

United States of America

Gianluigi Pasta

Italy

Lorenzo Pavone

Italy

Rinaldo Pellicano

Italy

Giorgio Perilongo

Italy

Wei Perng

United States of America

Serafina Perrone

Italy

Giorgio Piacentini

Italy

Claudio Pignata

Italy

Alessandro Plebani

Italy

Sergey Popov

United Kingdom

Carlotta Povesi Dascola

Italy

Kostas Priftis

Greece

Nikoleta Printza

Greece

Renato Procianoy

Brazil

Parthak Prodhan

United States of America

Valeria Raia

Italy

Francesco Raimondi

Italy

Barbara Rath

Germany

Russel Reiter

United States of America

Giampaolo Ricci

Italy

Miriam Rigoldi

Italy

Luis Rodrigo

Spain

Claudio Romano

Italy

Maria Grazia Roncarolo

Italy

Manuel Rosa-Fraile

Spain

Sergio Rosenzweig

United States of America

Mohammad Rostami Nejad

Iran

Bruce Rubin

United States of America

Franca Rusconi

Italy

Giovanna Russo

Italy

William Ryan

United States of America

Giuseppe Saggese

Italy

Amirhossein Sahebkar

Iran

Mariacarolina Salerno

Italy

Silvia Salvatore

Italy

Francesca Santamaria

Italy

Filippo M. Santorelli

Italy

Luca Scapoli

Italy

Maurizio Scarpa

Italy

Frieder Schaumburg

Germany

Annalisa Sechi

Italy

Angelo Selicorni

Italy

Ahmet Sert

Turkey

David Sheridan

United States of America

Roman Shypailo

United States of America

Jerry Simecka

United States of America

Olle Soder

Sweden

Gyula Soltesz

Hungary

Ahmet Soysal

Turkey

Martin Stocker

Switzerland

Maria E Street

Italy

Giovanna Stringari

Italy

Mauro Stronati

Italy

Linyan Su

China

Kazuysohi Suga

Japan

Scott Sutherland

United States of America

Tomoaki Taguchi

Japan

Fatih Tanriverdi

Turkey

Bertrand Tchana

Italy

Kadir Serafettin Tekgündüz

Turkey

Theoharis Theoharides

United States of America

Dick Tibboel

Netherlands

Liat Tikotzky

Israel

Brian Timmons

Canada

Emma Tippett

Australia

Omar Tliba

United States of America

Tullia Todros

Italy

Gina Tomé

Portugal

Sara Torretta

Italy

Iñigo Tuduri Limousin

Spain

Gulcan Turker

Turkey

Nicola Ullmann

Italy

Yoshihisa Urita

Japan

Eloisa Urrechaga

Spain

Vytautas Usonis

Lithuania

Pietro Vajro

Italy

Massimiliano Valeriani

Italy

Enrico Valletta

Italy

Sebastiaan Van As

South Africa

Koen Van Herck

Belgium

Margherita Varini

Ireland

Rajkumar Venkatramani

United States of America

Srinivasan Venkatramanujan

Malaysia

Maria Carmela Verga

Italy

Alberto Verrotti

Italy

Daniel Vijlbrief

Netherlands

Raffaele Virdis

Italy

Anju Virmani

India

Nicolas von der Weid

Switzerland

Stephen Wainer

Canada

Yehezkel Waisman

Israel

Anne Walsh

Australia

Jiang Huai Wang

Ireland

Jou-Kou Wang

Taiwan

Pornswan Wasant

Thailand

Alan Weindling

United Kingdom

Peter Weinstock

United States of America

Andrea Weintraub

United States of America

Derek Wheeler

United States of America

Yonatan Yeshayahu

Israel

Panayiotis Yiallouros

Cyprus

Zhangbin Yu

China

Ekhard Ziegler

United States of America

Jozsef Zsiros

Netherlands

Gian Vincenzo Zuccotti

Italy

Antonio Zuppa

Italy

